# Clinical and Therapeutic Notes

**Published:** 1887-11-05

**Authors:** C. R. Illingworth


### Clinical and Therapeutic Notes.

By C. R. Illingworth, M.D., M.R.C.S.

### I.—"FOR THE BLOOD IS THE LIFE."

A knowledge of the structure and functions of the blood, and of the possible changes in its composition, is essential to the formulation of any and every treatment of disease.

"The blood, as it flows from the living body, is seen to be a thickish, heavy fluid, of a bright scarlet colour when it comes from an artery; deep purple, or nearly black, when flowing from a vein. Although to the naked eye, however, it seems uniformly tinted, it is found by the microscope to be really a colourless fluid containing minute coloured cells or corpuscles; and these cells, which are red, when seen *en masse*, are the real source of the colour which seems to the naked eye to belong to every part of the blood alike. The coloured cells are termed blood cells, or *blood-corpuscles;* the colourless fluid portion is termed *liquor sanguinis*, or *plasma.*" (Kirkes.)

Shortly after being drawn from the body the blood forms a jelly-like mass or clot, due to the presence of *fibrin*. The exact cause of this coagulation or clotting of the blood is not known, but the phenomenon is generally believed to be due to the union of two elements, one of which is held by the corpuscles and named *globulin*, and the other by the plasma, and named *fibrinogen*.

From the surface of the clot a yellowish fluid exudes, sufficient in a little time, as the clot contracts and becomes firmer, to surround it. This fluid is named *serum*. Schmidt believes that the fibrin elements exist in the serum of the blood, and unite under certain circumstances to form fibrin, but he experimented with dropsical and lymphatic fluids only. Lister, who argues that blood has no natural tendency to clot, experimented with blood in its entirety, in order to demonstrate that its coagulation out of the body is due to the action of foreign matter with which it happens to be brought into contact, and in the body to abnormal conditions of the tissues in which their vital energies are prostrated by inflammation or injury. He ligatured the jugular vein of an ox in two places before death, cut out the capsule thus obtained, and hung it up by one end. The corpuscles sank to the bottom very rapidly, and formed a thick layer there, leaving the upper part transparent—the plasma of the blood pure and simple. He then punctured the upper portion of the capsule and drew off some of the fluid into a watch glass. It did not coagulate. But on the addition of a small piece of blood clot it immediately began to do so, by reason, he argued, of the union of the constituent of fibrine contained in the corpuscles entangled in the clot with the other constituent in the plasma. Fibrin was thus formed.

Now, inflammatory exudation represents plasma *plus* globulin; it contains both ingredients of fibrin because it is coagulable. It resembles the plasma of blood which has been shed from the living animal directly into a vessel, for coagulation takes place without any further interference with it or addition to it. In contradistinction to the inflamatory effusion, there is the œdema of a limb on ligature of the mainvein. The plasma here effused is uncoagulable, and givesrise to a swelling which pits on pressure, unlike the hard and brawny swelling of an inflamed limb. Müller experimented with frog's blood, by filtering it through some fine bibulous paper. He obtained a fluid which was clear, and coagulated after standing for a little time. From this he concluded that the fibrin of the blood must be dissolved in the plasma, because the corpuscles being arrested by the fine meshes of the paper, a colourless clot was obtained. Lister, however, maintains that the corpuscles had communicated their globulin to the plasma before it was filtered through the paper, which thus served a purpose similar to the walls of inflamed blood vessels or bruised tissues. Living solid matter therefore—as instanced by the experiment with the jugular vein of an ox—does not induce coagulation, *when it is healthy.* Healthy living tissues do not admit of the union of fibrin elements in their vicinity. In fact, blood has no spontaneous tendency to coagulate. It is only when acted upon by foreign substances, or by tissues, which, from imflamation or injury, have been reduced to a condition of prostration of their vital energy.

The uses of the blood need not be fully entered upon here. It will be sufficient to note that one of the chief is the conveyance of oxygen received during respiration, to all parts of the body. This process, as is well known, is effected by means of the colouring matter of the blood, known as hœmoglobin, which is capable of existing in two different states of oxidation, the one named scarlet hæmoglobin, containing most oxygen, and giving the red tint to arterial blood; the other purple, containing less oxygen, and giving the dark hue to venous blood.

The changes of the blood in disease relate chiefly to the increase or reduction of the amount of fibrin and hæmoglobin. Thus in all inflammatory conditions the fibrin is increased in amount. In acute articular rheumatism it has been found to be present in the proportion of 10·2 parts in 1,000, the normal amount being three to four parts. In pneumonia the proportion has been known to be 10·5. There is a deficiency of fibrin in all diseases proving fatal by asphyxia, such as suffocation, some forms of heart disease, drowning, and strangulation; also in purpura hœmorrhagica, and allied diseases. In anæmia or spanæmia there is deficiency of the hæmoglobin; whilst, in all diseases due to septicæmic processes, the blood is rapidly deprived both of fibrin and hœmoglobin, most probably owing to the depredations of micro-organisms peculiar to each disease in this class, upon these important constituents of the vital fluid.

(*To be continued.*)

